# A Solomonesque Command

**Published:** 1900-10

**Authors:** 


### A SOLOMONESQUE COMMAND.

THE Board of Health of East Orange, N. J., has been wrestling for months past with a most momentous question and has finally issued an edict which appears to have met with general approval.

East Orange is one of the prettiest spots in New Jersey, and is quite swell in its way, being populated largely by semi-aristocratic business people from New York and their families.

To live in the Oranges is to have a certain standing among the Jersey commuters, which is envied by the less fortunate individuals who are compelled for domestic reasons to get off at way stations. The population of the Oranges, too, do not carry bundles; their groceries and their other necessities are sent out by express. This explanation will give one an idea of the hauteur of the average resident of the Oranges; and one can the better understand the appalling position in which the East Orange found itself one fair morning in the recent past when complaints began to pour in regarding the noise made by domestic animals quartered in the rear of the palatial summer homes of the aristocratic habitues. Naturally, the Board of Health, made up of sensible-minded gentlemen—some of them undoubtedly had social ambitions—did not wish to openly offend Mrs. Harold Percival Smythe-Jounnes by announcing that the lowing of her cow disturbed the peaceful slumbers of Mrs. Alexander Aristotle Browne-Perkyns; nor did it wish to annoy Mr. Arthur Terwilliger-Terwilliger by ever so delicately calling his attention to Mr. Theodore Tudor's impertinent remonstrance that Mr. Theodore Tudor's beauty sleep was spoiled by the thoughtless kicking of Mr. Terwilliger-Terwilliger's horse.

So this East Orange Board of Health conferred and conferred and suggested and fretted and fumed, and at last a member, who is entitled by virtue of his wisdom to trace his ancestry back to the front door of Solomon's city home, unravelled the tangled social skein by offering a resolution which commanded that all domestic animals be kept quiet between 10 p.m. and 8 a.m.; and that in case of complaint, the animals be removed 200 feet from the complainant's house. Thus was the unruffled surface of society maintained in East Orange, and the beauty sleeps and the "forty-winks" may now be enjoyed in comfort and peace.

The dignity of the East Orange Board of Health has been maintained, but it must be a view for a kinetoscope, the spectacle of the East Orange people keeping their animals quiet between 10 p.m. and 8 a.m.

